# Retained fecalith following laparoscopic appendectomy

**DOI:** 10.1002/ccr3.3867

**Published:** 2021-01-27

**Authors:** Yegi Sandy Kim, Joseph Cherng Kong, Evan Williams, Satish K. Warrier

**Affiliations:** ^1^ Department of General Surgery Colorectal Surgery Unit Alfred Hospital Melbourne Vic Australia; ^2^ Division of Cancer Surgery Peter MacCallum Cancer Centre Melbourne Vic Australia; ^3^ Division of Cancer Research Peter MacCallum Cancer Centre Melbourne Vic Australia; ^4^ Sir Peter MacCallum Department of Oncology University of Melbourne Parkville Vic Australia

**Keywords:** laparoscopic appendectomy, pelvic abscess, retained fecalith

## Abstract

It is important to identify a retained fecalith and remove that infective nidus to decrease the morbidity in patients undergoing laparoscopic appendectomy for perforated appendicitis.

## Graphical abstract

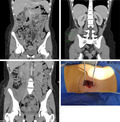

## 1 INTRODUCTION

There have been a few case studies showing intra‐abdominal abscesses due to retained fecalith post laparoscopic appendectomy. A 29‐year‐old woman presented with right lateral abdominal wall and pelvic collection due to retained fecalith post interval laparoscopic appendectomy for perforated appendicitis. She underwent exploration for fecalith retrieval successful outcome.

Laparoscopic appendectomy is currently the first choice of surgical approach for an acute appendicitis due to lower rate of surgical site infections, shorter hospital stay, and better cosmetic outcome.^1^, ^2^, ^3^ Furthermore, an interval appendicectomy is favored by many surgeons for treatment of perforated appendicitis. However, one of the complications from laparoscopic appendectomy includes intra‐abdominal abscess formation secondary to retained fecalith.^1^, ^4^ Although rare, a dropped fecalith can occur during appendectomy or due to expulsion from perforated appendix. We present a case of recurrent intra‐abdominal abscesses from a retained fecalith in the intramuscular layers of the iliacus in a patient whose initial diagnosis of an acute appendicitis was delayed.

## 2 CASE REPORT

A 29‐year‐old woman initially presented with a 2‐week history of nonmigratory right iliac fossa pain with subjective fever. The patient's history of complaint was not typical of acute appendicitis. After unremarkable inflammatory markers and pelvic ultrasound that did not visualize the appendix, she was discharged home with a provisional diagnosis of pelvic inflammatory disease and was given oral antibiotics for 7 days.

However, she represented to our emergency department (ED) with worsening abdominal pain, ongoing fever and mild nausea. A computed tomography (CT) was organized and it showed a perforated appendicitis with retrocecal abscess and a calcified appendicolith. She then had an ultrasound‐guided drainage of the abscess and was discharged home with a view of performing an interval laparoscopic appendectomy. After discharge, the patient returned to ED for persistent recurrence of a right pelvic collection involving the right iliacus and lateral abdominal wall muscle. This was again radiologically drained. A colonoscopy was performed during this presentation to exclude any primary tumors before surgery. The entire colon was examined and showed normal mucosa and appendiceal orifice.

**FIGURE 1:**
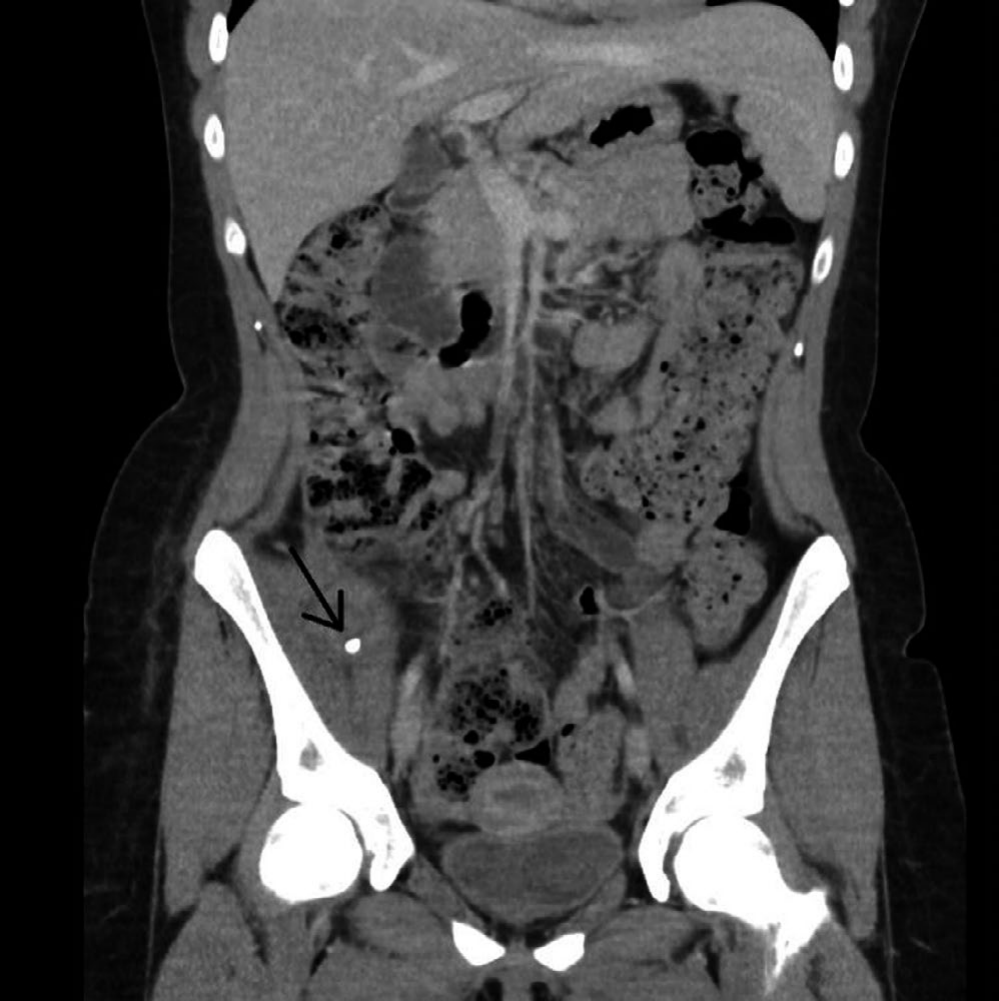
Computed tomography abdomen showing the retained fecalith

**FIGURE 2:**
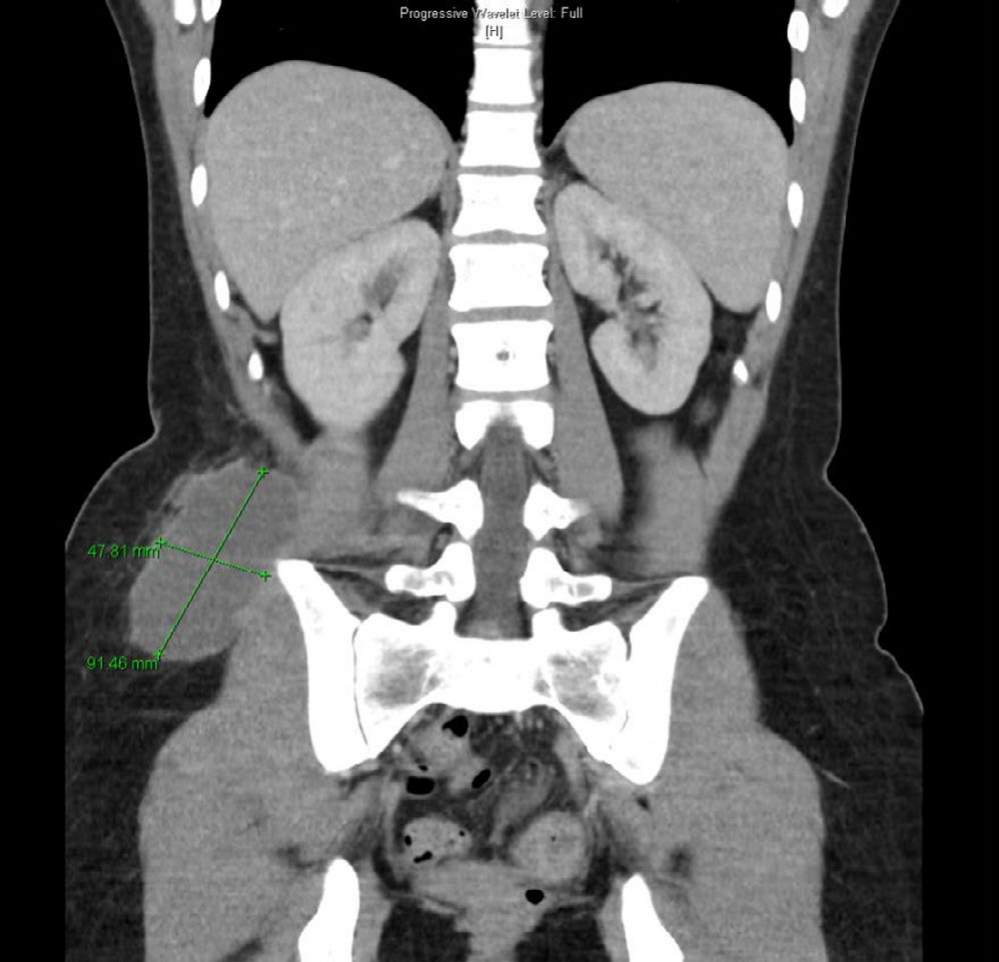
Computed tomography abdomen showing right lateral abdominal wall collection

The patient then underwent a semi‐emergent laparoscopic appendectomy. During the procedure, thorough abdominal wash was performed and a drain tube placed in the right iliac fossa. The patient had an uneventful postoperative recovery, with normalization of inflammatory markers and the drain tube removed. She was then discharged home with oral antibiotics. During her follow‐up in the general surgery clinic, the histopathology reported chronic appendicitis with granulomas but the fecalith was not within the specimen. On review, it was noted that she had intermittent abdominal discomfort and a repeat of C‐reactive protein (CRP) showed that it has elevated to 41. A repeat CT scan was performed which showed re‐accumulation of the right pelvic and lateral abdominal collection with the retained fecalith (Figures 1, 2).

**FIGURE 3:**
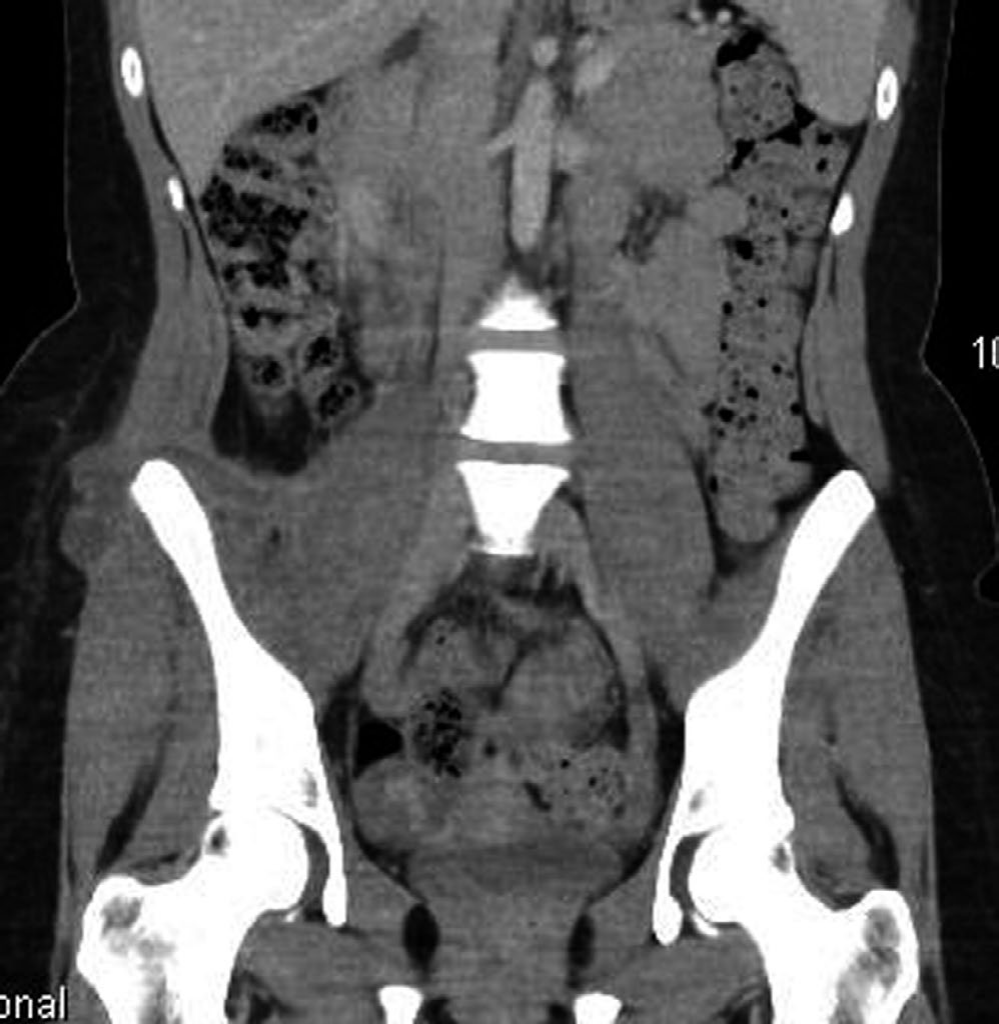
Computed tomography abdomen showing horseshoe shaped collection in right lateral abdominal wall into the subcutaneous fat

**FIGURE 4:**
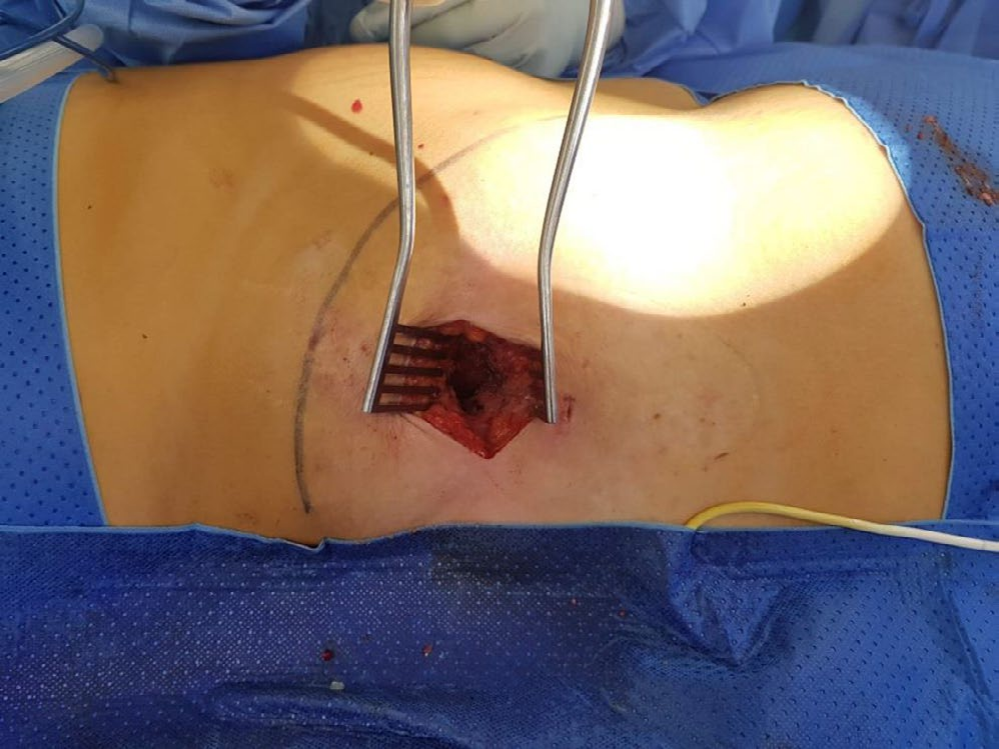
Intraoperative view of exploration

Although the abscess was intramuscular, the fecalith and the pelvic abscess were going through the abdominal wall into the subcutaneous fat (Figure 3). There was a brief discussion with an interventional radiologist for a hook‐wire insertion preoperatively for direct localization of the fecalith but given the location of the abscesses, intraoperative ultrasound was used instead. The patient underwent exploration and surgical removal of retained fecalith through a right lateral hip approach (Figure 4). A drain was inserted into the cavity and the wounds were closed with interrupted sutures.

## 3 OUTCOME/FOLLOW UP

The wound was complicated by formation of seroma, which was managed conservatively. The patient was reviewed again 6 weeks post‐operation and remained well with no further complications of her wound or pelvic abscess drainage.

## 4 DISCUSSION

An interval appendectomy for perforated appendicitis with established abscesses is widely practiced by many surgeons.^5^ It has been suggested that an interval appendectomy has many benefits including reducing the overall rate of adverse events.^6^ The concept of delaying surgery would allow the intraperitoneal contamination and inflammation to settle down with antibiotics, allowing surgeons to mitigate the risk of an unfriendly plane due to ongoing sepsis.^6^ However, the strategy of an interval appendectomy was not successful in our case due to an infective nidus, a retained fecalith. This particular complication, although not common, is a well‐known complication after a laparoscopic appendectomy. The risk of retained fecalith is even higher in a case of perforated appendicitis.^7^ The fecalith may drop from the base of the appendix when it is being resected or when the appendix is being extracted through the port.^7^, ^8^ A study suggests different strategies to prevent fecalith spillage including gentle manipulation of an appendix and use of an endoscopic bag to retrieve the appendix.^9^ We suspect that in our case the retained fecalith will likely have migrated from the perforated appendix into the intramuscular layers of iliacus post percutaneous drainage of the abscess.

There have been different recommendations for the management of a retained fecalith.^1^, ^10^ Black et al^4^ presented a case of an abscess with fecalith adequately managed with intravenous antibiotics only. Some studies suggest percutaneous drainage and extraction of fecalith.^4^, ^10^ Despite these, surgical removal of fecalith is recommended by many surgeons.^1^ We had two rationales for a right lateral hip approach instead of intra‐abdominal approach. Firstly the location of the abscess was continuous from subcutaneous plane to intra‐abdominal plane. Secondly the planes of dissection and retrieving the fecalith would be difficult if through intra‐abdominal. Hence, taking a new approach through virgin territory has aid with the recovery of the fecalith. Although she developed seroma post operatively, she recovered relatively quickly after the operation.

This case highlights the importance of identifying and extracting a retained fecalith to decrease the morbidity associated with a persistent infective nidus. Given that the patient had a laparoscopic procedure with a failed attempt, we have decided on a different surgical access, right lateral hip approach, with the intention of a midline laparotomy if not successful. This has led to a successful resolution of her right pelvic abscess formation with no added surgical morbidity.

## CONFLICT OF INTEREST

There are no conflicts of interest.

## AUTHOR CONTRIBUTIONS

YSK: have made substantial contribution to conception of the manuscript; Been involved in drafting, writing and revising the manuscript; Obtaining patient consent, administrative work. JCK: have made substantial contribution to conception of the manuscript; Proof‐reading, writing assistant; Final approval for publication. EW: proof‐reading; Language editing; Final approval for publication. SKW: general administrative support; Proof‐reading; Final approval for publication.

## ETHICAL APPROVAL

Patient's informed and signed consent obtained.

## Data Availability

Data sharing not applicable to this article as no datasets were generated or analyzed during the current study.
